# Supplementary material for: Empirical methods for controlling false positives and estimating confidence in ChIP-Seq peaks
Source: BMC Bioinformatics. 2008 Dec 5;9:523. doi: 10.1186/1471-2105-9-523 (PMC2628906; doi:10.1186/1471-2105-9-523)
Supplement: Additional File 1 — A variety of html documents from the USeq web site detailing the available applications, their best usage, output file type descriptions, command line menus, etc. [file 1471-2105-9-523-S1.zip › USeqUserGuides/JavaDoc.zip!/JavaDoc/trans/bkgrnd/class-use/BackGroundOligo.html]

Uses of Class trans.bkgrnd.BackGroundOligo


|  |  |  |
| --- | --- | --- |
| **Overview** \| **Package** \| **Class** \| **Use** \| **Tree** \| **Deprecated** \| **Index** \| **Help** | |  |
| PREV   NEXT | **FRAMES**    **NO FRAMES**     **All Classes** |  |


---


## **Uses of Class trans.bkgrnd.BackGroundOligo**

| Packages that use BackGroundOligo | |
| --- | --- |
| **trans.bkgrnd** | For examining background GC content and ratio bias. |

Uses of BackGroundOligo in trans.bkgrnd

| Methods in trans.bkgrnd that return BackGroundOligo | |
| --- | --- |
| `static BackGroundOligo[]` | `MapControlIntensities.makeInitialBGOs(java.io.File bpmap)` |

| Methods in trans.bkgrnd with parameters of type BackGroundOligo | |
| --- | --- |
| `static void` | `MapControlIntensities.loadIntensities(BackGroundOligo[] oligos, java.io.File[] celFiles)` |

---


|  |  |  |
| --- | --- | --- |
| **Overview** \| **Package** \| **Class** \| **Use** \| **Tree** \| **Deprecated** \| **Index** \| **Help** | |  |
| PREV   NEXT | **FRAMES**    **NO FRAMES**     **All Classes** |  |


---
